# Supplementary material for: Breastfed and Formula-Fed Infants: Need of a Different Complementary Feeding Model?
Source: Nutrients. 2021 Oct 24;13(11):3756. doi: 10.3390/nu13113756 (PMC8624840; doi:10.3390/nu13113756)
Supplement: Supplementary file 1 [file nutrients-13-03756-s001.zip › nutrients-1388665-supplementary.pdf]

Menù 6-8 mesi

Alimenti		Gr	Kcal	P
Latte materno		688	467,84	6,192
cereali		25	88,75	2,5
olio evo		10	89,9	
verdure		20		0,4
vitello		10	10,7	2,07
frutta		40	16,8	0,28
	TOTALE		673,99	11,442
	KCAL		671,613	45,768
	%	più8% rispetto a 622		6,81
8,08Kg 6-9 mesi EFSA	g/kg			1,42
	PRI			1,3g/Kg/die

EFSA 77kcalorie /kg/die= 622

L	L sat.	CHO	Zuccheri	Fibra	Fe	Ca	Na
24,08	10,8016	55,04	55,04		0,4128	158,24	
0,325	0,075	18,55			2,4	48,75	
9,99					0,02		
0,06		0,56	0,52	0,38	0,18	9	8,4
0,27	0,12				0,12	1,4	8,9
0,08		4	3,92	1,04	0,16	9,2	1,04
34,805	10,9966	78,15	59,48	1,42	3,2928	226,59	18,34
313,245		312,6					
46,64		46,54					

Menù 6-8 mesi

**Alimenti**		**Gr**	**Kcal**	**P**	**L**
F2		688	464,4	10,32	22,0848
cereali		25	88,75	2,5	0,325
olio evo		10	89,9		9,99
verdure		20		0,4	0,06
vitello		10	10,7	2,07	0,27
frutta		40	16,8	0,28	0,08
	TOTALE		670,55	15,57	32,8098
	KCAL		674,2962	62,28	295,2882
	%			9,24	43,79
8,08Kg 6-9 mesi EFSA	g/kg			1,93	
	PRI			1,3g/Kg/die	

L sat.	CHO	Zuccheri	Fibra	Fe	Ca	Na
8,6688	56,072	41,6928		6,8112	481,6	
0,075	18,55			2,4	48,75	
				0,02		
	0,56	0,52	0,38	0,18	9	8,4
0,12				0,12	1,4	8,9
	4	3,92	1,04	0,16	9,2	1,04
8,8638	79,182	46,1328	1,42	9,6912	549,95	18,34
	316,728					
	46,97					

Menù 18 mesi

**Alimenti**		**Gr**	**Kcal**	**P**	**L**
F3		488	295,728	8,1008	12,688
pasta		30	102,3	4,05	0,36
Petto di pollo		20	20	4,66	0,16
olio evo		20	179,8		19,98
verdure		60		1,2	0,18
riso		30	100,2	2,01	0,12
piselli		30	18,3	1,62	0,09
frutta		150	63	1,05	0,3
	TOTALE		779,328	22,6908	33,878
	KCAL		772,9772	90,7632	304,902
	%			11,74	39,45
10,55Kg 18 mesi EFSA	g/kg			2,15	
	PRI			1-1,3g/Kg/die	

**L sat.**	**CHO**	**Zuccheri**	**Fibra**	**Fe**	**Ca**	**Na**
4,148	29,768	29,28		4,88	400,16	
0,06	21,81	0,66	0,51	0,42	6,6	1,2
0,06				0,08	0,8	6,6
				0,04		
	1,68	1,56	1,14	0,54	27	25,2
0,03	24,12	0,06	0,3	0,24	7,2	1,5
	1,95	1,2	1,89	0,6	6	38,7
	15	14,7	3,9	0,6	34,5	3,9
4,298	94,328	47,46	7,74	7,4	482,26	77,1
	377,312					
	48,81					

Menù 18 mesi

**Alimenti**		**Gr**	**Kcal**	**P**	**L**
LV		488	312,32	16,10	17,57
pasta		30	102,30	4,05	0,36
Petto di pollo		20	20,00	4,66	0,16
olio evo		20	179,80		19,98
verdure		60		1,20	0,18
riso		30	100,20	2,01	0,12
piselli		30	18,30	1,62	0,09
frutta		150	63,00	1,05	0,30
	TOTALE		795,92	30,69	38,76
	KCAL		825,49	122,78	348,82
	%			14,87	42,26
10,55Kg 18 mesi EFSA	g/kg			2,91	
	PRI			1-1,3g/Kg/die	

L sat.	CHO	Zuccheri	Fibra	Fe	Ca	Na
10,25	23,91	23,91		0,49	580,72	
0,06	21,81	0,66	0,51	0,42	6,60	1,20
0,06				0,08	0,80	6,60
				0,04		
	1,68	1,56	1,14	0,54	27,00	25,20
0,03	24,12	0,06	0,30	0,24	7,20	1,50
	1,95	1,20	1,89	0,60	6,00	38,70
	15,00	14,70	3,90	0,60	34,50	3,90
10,40	88,47	42,09	7,74	3,01	662,82	77,10
	353,89					
	42,87					

Menù 18 mesi

**Alimenti**		**Gr**	**Kcal**	**P**	**L**
LM		488	331,84	4,392	17,08
pasta		30	102,3	4,05	0,36
Petto di pollo		20	20	4,66	0,16
olio evo		20	179,8		19,98
verdure		60		1,2	0,18
riso		30	100,2	2,01	0,12
piselli		30	18,3	1,62	0,09
frutta		150	63	1,05	0,3
	TOTALE		815,44	18,982	38,27
	KCAL		834,758	75,928	344,43
	%			9,10	41,26
10,55Kg 18 mesi EFSA	g/kg			1,80	
	PRI			1-1,3g/Kg/die	

L sat.	CHO	Zuccheri	Fibra	Fe	Ca	Na
7,6616	39,04	39,04		0,2928	112,24	
0,06	21,81	0,66	0,51	0,42	6,6	1,2
0,06				0,08	0,8	6,6
				0,04		
	1,68	1,56	1,14	0,54	27	25,2
0,03	24,12	0,06	0,3	0,24	7,2	1,5
	1,95	1,2	1,89	0,6	6	38,7
	15	14,7	3,9	0,6	34,5	3,9
7,8116	103,6	57,22	7,74	2,8128	194,34	77,1
	414,4					
	49,64					
